# Standardization of an experimental model of intradural injection after spinal cord injury in rats

**DOI:** 10.6061/clinics/2021/e2740

**Published:** 2021-03-19

**Authors:** Olavo B. Letaif, Mauro C.M. Tavares-Júnior, Gustavo B. dos Santos, Ricardo J.R. Ferreira, Raphael M. Marcon, Alexandre F. Cristante, Tarcísio E.P. de Barros-Filho

**Affiliations:** Departamento de Ortopedia e Traumatologia, Instituto de Ortopedia e Traumatologia, Hospital das Clinicas HCFMUSP, Faculdade de Medicina, Universidade de Sao Paulo, Sao Paulo, SP, BR.

**Keywords:** Spinal Cord Injury, Rats, Animal Models, Intrathecal Route

## Abstract

**OBJECTIVES:**

The intrathecal route has not yet been thoroughly standardized and evaluated in an experimental model of spinal cord injury (SCI) in Wistar rats. The objective of this study was to standardize and evaluate the effect of intradural injection in this animal model.

**METHOD:**

The animals were divided into 6 groups: 1) laminectomy and intradural catheter; 2) laminectomy, intradural catheter and infusion; 3) only SCI; 4) SCI and intradural catheter; 5) SCI, intradural catheter and infusion; and 6) control (laminectomy only). Motor evaluations were performed using the Basso, Beattie and Bresnahan (BBB) scale and the horizontal ladder test; motor evoked potentials were measured for functional evaluation, and histological evaluation was performed as well. All experimental data underwent statistical analysis.

**RESULTS:**

Regarding motor evoked potentials, the groups with experimental SCI had worse results than those without, but neither dural puncture nor the injection of intrathecal solution aggravated the effects of isolated SCI. Regarding histology, adverse tissue effects were observed in animals with SCI. On average, the BBB scores had the same statistical behaviour as the horizontal ladder results, and at every evaluated timepoint, the groups without SCI presented scored significantly better than those with SCI (*p*<0.05). The difference in performance on motor tests between rats with and without experimental SCI persisted from the first to the last test.

**CONCLUSIONS:**

The present work standardizes the model of intradural injection in experimental SCI in rats. Intrathecal puncture and injection did not independently cause significant functional or histological changes.

## INTRODUCTION

Spinal cord injury (SCI) originates from accidents or violence and mainly affects young adults. Although the incidence of SCI is usually less than 50 per million, the associated morbidity and mortality are costly, with hospital expenses of approximately US$95,000 resulting from initial hospitalization. In addition, there are generally important motor sequelae and loss of function, with 45% of the lesions being complete (1). The estimated global incidence rate is 23/million inhabitants, with a prevalence between 236 and 4187/million inhabitants (2).

After SCI, functional loss results from a two-stage process: primary injury, involving cell death due to mechanical stress, and secondary injury, associated with a series of neurochemical alterations (3). Because primary injury is irreversible, therapeutic efforts focus on reducing secondary injury, decreasing inflammation, and promoting axonal regeneration. Experimental investigations have suggested corticosteroids (4), estrogen (5), progesterone (6), selective serotonin reuptake inhibitor antidepressants (7), and other substances as possible therapeutic agents.

In the study of SCI, experimental animal models, in addition to facilitating research on the effects of different drugs, also allow the analysis of different routes for the administration of these drugs (8). Different pathways can have distinct advantages, disadvantages and complications (8,9). There is no reliable basis in the literature to state that one route of administration is definitely superior to the other in SCI (8). The intrathecal route (IR) has benefits such as technical simplicity and low tissue damage (10,11). The IR can be used to administer both medicines (12-15) and cells, such as stem cells (16-20).

The experimental rat model of SCI is well established (21), but the administration of drugs through the IR in this model is not. The standardization, evaluation, safety (22) and reproducibility of the IR for research have not yet been studied in full, considering the motor, functional and histological aspects (23,24). The present study aims to standardize the use of the IR in rats subjected to controlled experimental SCI. Another objective of this research is to measure the functional and histological effects of fluid infusion in the intradural space at the site of medullary tissue contusion, since there is little literature on these effects (25,26). The establishment of this protocol will bring expanded research perspectives (27,28) by establishing a standardized model for the intrathecal administration of substances.

## MATERIALS AND METHODS

This research protocol (number 1222) was evaluated and approved by the Scientific Committee of the IOT and the Ethics Committee for The Analysis of Research Projects of the Hospital das Clínicas of FMUSP.

This study was conducted at the Institute of Orthopedics and Traumatology (IOT) of the Faculty of Medicine of the University of São Paulo (FMUSP).

A total of 48 rats were included, with 8 individuals per group, which is similar to the group sizes used in previous studies (8,16). The following inclusion criteria were adopted: Wistar rats; young adult males (mean age of 20 weeks); weight between 340g and 450g; good general condition; normal initial motricity. The exclusion criteria were as follows: death after experimental SCI; persistent infection even after 10 days of antibiotic therapy; macroscopically observed anomalies of the medullary region; autophagy or mutilation; and loss of more than 10% of body weight after SCI. The rats were kept in cages stored on metabolic racks under controlled conditions.

For the formation of the experimental groups, which were followed for 6 weeks, the animals were randomly divided into 6 groups of 8 animals each:

- Group 1: Laminectomy + intradural catheter introduction (ICI);
- Group 2: Laminectomy + ICI + infusion of 0.1 ml of saline solution (ISS);
- Group 3: Laminectomy + SCI;
- Group 4: Laminectomy + SCI + IC;
- Group 5: Laminectomy + SCI + ICI + ISS;
- Group 6: Control (sham) group that underwent only a laminectomy at the same anatomical level as the other groups.

Basso, Beattie and Bresnahan (BBB) scale, sensorimotor behavioral evaluation: horizontal ladder, motor evoked potential (MEP) and histology data analyses were performed in a blinded manner. However, both the principal investigator and the veterinarian were aware of each animal group.

Anaesthesia was induced with isoflurane, and after mild sedation was reached, a mask was placed over the snout of the animal to achieve a deeper plane of anaesthesia (21). Then, the hair was removed from the back, and the region was cleaned.

All SCI procedures were performed using an NYU impactor (21), which dropped a mass of 10 g from a height of 12.5 mm (mild contusion). For this purpose, a laminectomy was performed, exposing the spinal cord in a standardized manner. A midline incision was made in the skin to expose T8 - T12. The laminae and spinous processes of T9 and T10 were removed. The laminar opening was sufficient to accommodate the head of the impact rod device and to promote SCI (26).

With the rats still anaesthetized, the experimental SCI complete, and the thoracic laminectomy site still exposed, the animals of groups 1, 2, 4 and 5 were submitted to intradural puncture and ICI.

**Figure 1:**
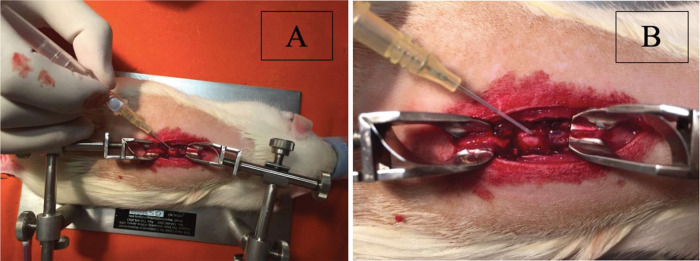
Center of laminectomy. **A)** Puncture of the dural sac at the center of laminectomy at the site of experimental spinal cord contusion. **B)** Needle removal and intrathecal introduction of the catheter approximately 5 mm in length.

Dural sac puncture was performed at the site of the experimental medullary contusion in the centre of the laminectomy. For puncture, a needle and a peripheral intravenous catheter assembly from B. Braun (Introcan^®^ Safety™: 4251601-04) were used. The needle had a size (calibre) of 24 G, and the catheter had a length of 3/4 (three-quarters) of 19 mm, with a diameter of 0.7 mm; the catheter flow was 22 ml/minute, or 1320 ml/hour (Figure 1A).

After the puncture, the needle was carefully withdrawn from the dural sac, and, with continued caution, the catheter was introduced intradurally for approximately 5 mm inside the intrathecal space. After the safety of the catheter position was assured, 0.1 ml of saline solution was injected intrathecally in groups 2 and 5 (Figure 1B).

In groups 1, 2, 4 and 5, the catheter was removed after its introduction, and at that moment, the regularity and closure of the defect produced in the dural sac and the eventual output of cerebrospinal fluid through the puncture hole were observed.

After surgery, all the rats were given antibiotic prophylaxis and pain relief medication. After SCI, the animals lost the urination reflex; accordingly, the bladder was emptied manually (7,21). The rats were returned to their original cages and were kept there under the same environmental conditions until the end of the experiment. During the evaluation period, rats were observed for mutilation, infections or other alterations.

### Protocol for assessing locomotor capacity on the Basso, Beattie and Bresnahan (BBB) scale

Locomotor capacity after SCI was measured on the BBB functional evaluation scale (29). The BBB is the main scale used to quantify motor recovery in rats with SCI in studies conducted by MASCIS (Multicenter Animal Spinal Cord Injury Study) (29).

All rats in the groups were evaluated by the BBB scale on the 2^nd^, 7^th^, 14^th^, 21^st^, 28^th^, 35^th^ and 42^nd^ days after SCI, and each rat′s evaluation was simultaneously performed by two observers, adequately trained, who did not know the group assignment of the rat; the observers were also blinded to each other′s evaluations to avoid interference. If there was disagreement between the evaluations, the lowest score was recorded.

### Sensorimotor behavioural evaluation: horizontal ladder

A horizontal ladder 100 cm long, 35 cm wide, suspended 46 cm from the ground, with a fixed space of 1.5 cm between the iron rungs, was used to evaluate the proprioceptive function of the animals (30). The animals were first trained to walk on the ladder for two days before the surgical experiments they were required to cross it five times. In the postoperative evaluation, the animals were required to walk voluntarily along the ladder, and the numbers of total steps, correct steps, slipping steps and errors were recorded.

Correct steps consisted of correct positioning of the paws on the rungs. The positioning of the paws on the rung, followed by a fall between the rungs, were considered to be slipping steps. Two types of errors were considered separately, namely, the dragging of the hind limbs along the horizontal ladder and the positioning of the paws between the iron rungs. The values of the three passages through the horizontal ladder were obtained for all types of answers (correct steps, slipping steps and errors), and they were averaged.

### Motor evoked potential (MEP) analysis

On the 42^nd^ postoperative day, the rats were once again anesthetized to perform the MEP test to record the values of amplitude and latency of response in the paws after transcranial electrical stimulus (5). The capture of muscle responses was performed with standardized methodology (5).

### Euthanasia protocol

At the end of the trial period (42 days), all rats were euthanized according to current legislation and following the precepts of the Brazilian College of Animal Experimentation.

The animals were placed in a chamber with halothane to promote mild sedation, followed by a lethal intraperitoneal dose of ketamine and xylazine. They were killed painlessly with thiopental and intravenous potassium chloride.

### Necroscopic and pathological examination

Necroscopic examinations were conducted on the rats to allow macroscopic identification of possible alterations. The presence of lesions associated with autophagy or mutilation was observed.

The internal inspection initiated with the removal of the spine, with an extensive dorsal incision. A spine segment was cut from T8 to T12 (including the area of SCI). All bone and soft tissue structures adjacent to the spinal cord were removed until it was fully exposed. A macroscopic visual evaluation was performed at the contusion site to verify any anomaly (exclusion criterion).

### Histological evaluation

The specimen of the sectioned spinal cord obtained for histological analysis was fixed linearly on cardboard with the appropriate topographic identifications. Where the macroscopic findings of SCI were observed, this region was identified as area “B”; the areas cranial to the lesion were designated “A”, and the areas caudal to the lesion were designated “C”.

Each area previously identified as “A”, “B” and “C” was sectioned in the axial plane at intervals of 2 mm, starting from area “A”. Each fragment was blocked in paraffin in a standardized fashion and later identified with the topography of the respective material. The paraffin blocks were sent to the microtomy process using a microtome and disposable slides. Transverse and sagittal sections of the spinal cord at the epicentre of the lesion and adjacent areas were performed. The slides were stained with haematoxylin and eosin.

**Figure 2:**
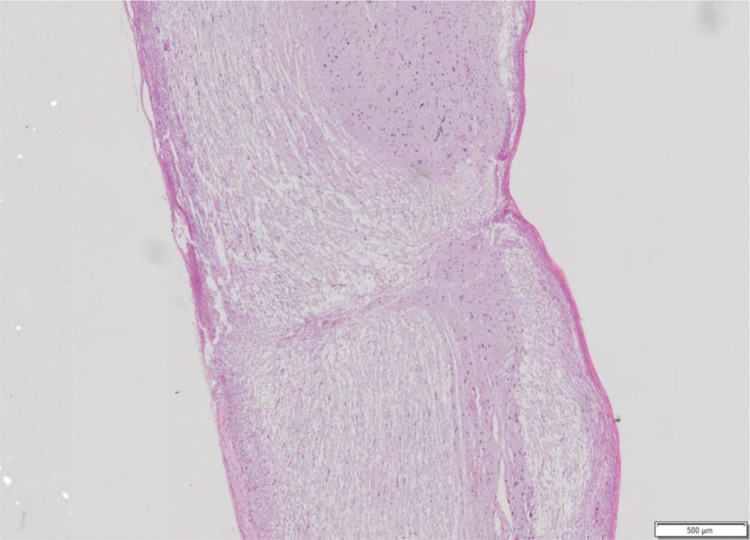
Spinal cord fragment. Sagittal cutting microscopy of spinal cord fragment, animal 7 of group 4. Hematoxylin-eosin staining showing histological features with almost complete interruption of neural bundles, hemorrhage (1+), architectural changes (3+), necrosis (2+) and inflammatory infiltrate (1+). Photo obtained using the Olympus scanner, model VS120, 20x lens.

The following variables were observed: haemorrhage, architectural alterations, necrosis, and cellular inflammatory infiltrate. For each variable, a score from 0 to 3 was assigned, where 0 is absent, 1 is discrete, 2 is moderate, and 3 is intense, to allow statistical analysis (Figure 2).

### Analysis of results

This study′s aim is to evaluate differences between the groups in the parameters evaluated and throughout the follow-up, when applicable.

Initially, a t-test was performed to evaluate whether the scores applied (BBB motor test) and the values found (MEP) referring to the paws (right and left) belonging to the same rat were equivalent. The t-test showed that the paws of each rat presented similar scores and values. Thus, the score or value of each individual was included as that corresponding to the arithmetic mean of their paws.

The single-timepoint characteristics, MEP and histology were described according to groups using summary measures (mean, standard deviation, median, minimum and maximum) and compared between groups using analysis of variance (ANOVA), followed by multiple comparisons with the Bonferroni correction when significant, or by the Kruskal-Wallis test, followed by Dunn's multiple comparison when significant.

The motor tests were described according to groups throughout the evaluation moments with the use of summary measures and compared between the groups and moments of evaluation. For this, generalized estimation equations were used, with normal marginal distribution and identity link function, with matrix of correlations between the moments of self-regressive evaluation of the first order. When the analyses found significant results, they were followed by multiple comparisons with the Bonferroni correction to identify the groups or timepoints between which the differences occurred.

The analyses were performed using IBM-SPSS for *Windows* version 22.0 software, using Microsoft-Excel 2010 software, and tests were performed with a significance level of 5%.

## RESULTS

Table 1 shows that lower limb (LL) latency was on average higher in group 2 than in the other groups (*p*<0.05) and was lower in group 6 than in the other groups (*p*<0.05). The amplitude of LL was statistically higher in group 1 than in the other groups (*p*<0.001), except for group 6, whose LL amplitude was statistically higher than in the other groups (*p*<0.001).

Table 2 shows that for all histologically evaluated parameters, groups 3 and 4 presented statistically higher values than groups 6, 1 and 2 (*p*<0.05).

Table 3 shows that in the groups with SCI, there was a mean reduction in the values of the horizontal ladder in the comparison of the preoperative values for the other moments evaluated (*p*<0.001). In these groups, the values increased on average in the evaluation at 6 weeks in relation to the evaluation at 2 days and 3 weeks (*p*<0.001).

Table 4 shows that only in the groups with SCI was there a statistically significant mean change in the BBB score between the moments evaluated (*p*<0.05), and the BBB score increased on average over time in the groups with SCI.

## DISCUSSION

This work′s objectives are to evaluate the effect of injection and volume administration of intradural solution on SCI in Wistar rats and standardize this experimental model through the analysis of its safety and reproducibility.

This research design aims to demonstrate that the tissue trauma caused by needle placement and catheter placement in the intradural space, together or not with ISS, is not an independent factor of negative interference in the histological effects and functional performance of individuals. The sample consisted of 48 rats, a number similar to previous studies (8,16) and enough to give power to the analysis of the results. The division into six groups aims precisely to experimentally isolate the intrathecal injection′s (puncture) and volume administration′s effects from the experimental SCI′s effects (21).

Group 6 has the best performance in the analysis of MEP′s results. In group 2, good responses from both upper limbs and LL were expected, since there was no SCI; however, in group 2, LL latency was increased. This finding may be interpreted as originating from an anaesthetic or technical factor. Group 1′s results were slightly worse than group 6′s results, possibly indicating that the introduction of the intradural needle at the SCI′s site may have been a cause of disturbance in electrophysiological medullary functioning, even if slightly. In groups 3, 4 and 5, increased latency and reduced amplitude were observed in the LL. However, in the comparison between groups 3, 4 and 5, there was no statistically significant difference. It is possible to infer, therefore, that neither puncture nor ISS aggravated the isolated experimental SCI outcomes.

In the histological comparison, groups 3 and 4 presented statistically higher values than groups 1, 2 and 6 (*p*<0.05). The values found for group 5 were not as high as expected, including no significant difference for groups without SCI. In group 5, there was also no difference with the other two groups that suffered SCI. A possible explanation for this finding is that the group 5′s results come from a tissue sample selection near the SCI epicentre but not exactly from the epicentre. The results in groups 1 and 2 when compared to group 6 stand out. In this comparison, there was no statistically significant difference in any of the histological parameters evaluated, which means that after 6 weeks of needle introduction into the intradural space, there were no significant histological alterations.

From 2 days on, the values obtained for the horizontal ladder were statistically higher in groups 1, 2 and 6 than in groups 3, 4 and 5 (*p*<0.05), thus forming two groups of animals, those that suffered SCI and those that did not. This difference remains from the first to the last evaluation. In groups 1 and 2, when compared to group 6, there was no statistically significant difference at any time of motor evaluation. This means that the intradural space puncture and the ISS did not cause significant motor repercussions, neither acute nor chronic. In the groups with SCI, there was an average reduction in the values found throughout the experimental period in relation to the initial preoperative values (*p*<0.001). However, in these same groups, the values obtained on average increased again 6 weeks after SCI compared to 2 days and 3 weeks (*p*<0.001). The observed late improvement can be explained by spinal cord areas that have recovered over time, generating some degree of motor recovery, as already observed by previous studies (5,7).

The BBB score presented the same behaviour as the horizontal ladder, and at all evaluated times, the groups without SCI presented statistically higher scores than the groups with SCI (*p*<0.05). The difference remained between rats with or without SCI from the first to the last test. No statistically significant difference was observed at any time of the motor evaluation between groups 1 and 2 when compared to group 6. This finding reinforces that intradural space puncture and ISS do not cause significant motor repercussions, either acute or chronic. Only in the groups with SCI was there a statistically significant mean change in the BBB score along the evaluated moments (*p*<0.05). The late improvement in BBB test results in individuals with SCI has been previously described by past studies (5,7).

To evaluate IR use, there are two main points of doubt and possible confusion. The first concern is the tissue lesion caused by needle puncture in the meninge and intradural catheter introduction. It is imperative that this trauma does not interfere in the objective analysis of what is being primarily evaluated (17). The second point refers to the administration of a given volume of intrathecal solution. It should be ensured that the solution′s volume infused is not independently deleterious, whether interference is caused by mechanical, inflammatory or toxic effects (12,22). These two points should not lead to outcome changes in the experimental SCI model (13,14,16), which alone causes known motor, functional and histological repercussions (21). It is also of paramount importance that the only observed effects are those directly related to the evaluated experimental therapeutic intervention.

Unlike precursor studies with chronic intrathecal space catheterization (9,11), the present study used the catheter only during the time of ISS. Avoiding the maintenance of the catheter within the intradural space eliminates the factor of direct interference of the catheter′s presence on functional and histological outcomes. Thus, the single and immediate use of the catheter exempts it from being considered a confusion factor in the interpretation of the results.

The experimental results found in this study reinforce previous studies′ findings (22-24), which show the safety and reproducibility of the IR for research. However, these previous studies did not individually evaluate the IR in its entirety, considering motor, functional and histological aspects in the same assay. In this sense, the present study is a pioneer in the joint analysis of two motor functional tests, MEP and histological evaluation in an SCI animal model associated with intrathecal injection.

In the present work, there are some limitations. One of them is that we did not evaluate acute histological changes. Possible acute differences may be better studied if animal sacrifice occurs earlier, as performed by different authors (20). Furthermore, regarding the experiment′s time interval, the current work did not perform as in previous studies (19,20) that performed intradural punctures in different time phases after experimental SCI; it is possible that intradural puncture has different histological and functional repercussions depending on the temporal inflammatory phase in which the SCI is found. It is also this study′s limitation, the performance of intradural puncture only in the area of the SCI′s epicenter (24), differently from other authors (18) that published better functional recovery with intrathecal stem cells given distally to the anatomical site of SCI.

The present study standardizes the use of the IR in rats submitted to controlled, experimental SCI and measures the functional and histological effects resulting from the puncture and infusion of fluid in the intradural space at the SCI site. The already established animal model of experimental SCI in rats (21) may have its employment expanded using the IR for the administration of drugs and substances and in the study of cell therapy. The mastery of this technique provides the possibility of expanding the research protocols and the range of experimental therapeutic interventions (27,28).

## CONCLUSIONS

The present work standardizes the experimental model of intradural injection in SCI in rats. Through the evaluation of motor functional parameters, MEP and histology, it demonstrates the reproducibility and, fundamentally, the safety of dural puncture and intrathecal injection in animals with experimental SCI. Puncture and intrathecal injection did not independently cause significant functional or histological changes in the study animals.

## AUTHOR CONTRIBUTIONS

Leitaf OB designed the study, collected and analyzed the data, performed the procedures, wrote and reviewed the manuscript final version. Tavares-Júnior MCM reviewed the manuscript final version. Santos GB analyzed the data and literature, performed the procedures, and reviewed the manuscript. Ferreira RJR performed the final review of the literature and project, analyzed the data, performed the procedures, and reviewed the manuscript. Marcon RM and Cristante AF performed the final review of the literature and project, analyzed the data, and reviewed the manuscript. Barros-Filho TEP performed the final review of the literature and project, designed the study, analyzed the data, and reviewed the final version of the manuscript.

## Figures and Tables

**Table 1:** Comparison of the parameters of the evoked potential in two-by-two group comparisons.

							CI (95%)	
Variable	Comparison		Mean Difference	Standard Deviation	*p*	Inferior	Superior
Latency MS	Laminectomy + Catheter *vs*	Laminectomy + Catheter + Infusion	1.35	0.45	0.076	-0.07	2.77
	Laminectomy + Catheter *vs*	Medullar Contusion	-0.23	0.44	>0.999	-1.61	1.15
	Laminectomy + Catheter *vs*	Contusion + Catheter	-0.24	0.45	>0.999	-1.67	1.18
	Laminectomy + Catheter *vs*	Contusion + Catheter + Infusion	0.04	0.45	>0.999	-1.38	1.47
	Laminectomy + Catheter *vs*	Laminectomy	0.33	0.44	>0.999	-1.05	1.71
	Laminectomy + Catheter + Infusion *vs*	Medullar Contusion	-1.58	0.44	**0.014**	-2.96	-0.20
	Laminectomy + Catheter + Infusion *vs*	Contusion + Catheter	-1.59	0.45	**0.018**	-3.02	-0.17
	Laminectomy + Catheter + Infusion *vs*	Contusion + Catheter + Infusion	-1.31	0.45	0.098	-2.73	0.12
	Laminectomy + Catheter + Infusion *vs*	Laminectomy	-1.02	0.44	0.390	-2.40	0.36
	Medullar Contusion *vs*	Contusion + Catheter	-0.01	0.44	>0.999	-1.39	1.37
	Medullar Contusion *vs*	Contusion + Catheter + Infusion	0.27	0.44	>0.999	-1.10	1.65
	Medullar Contusion *vs*	Laminectomy	0.56	0.42	>0.999	-0.77	1.89
	Contusion + Catheter *vs*	Contusion + Catheter + Infusion	0.29	0.45	>0.999	-1.14	1.71
	Contusion + Catheter *vs*	Laminectomy	0.57	0.44	>0.999	-0.80	1.95
	Contusion + Catheter + Infusion *vs*	Laminectomy	0.29	0.44	>0.999	-1.09	1.67
Latency MI	Laminectomy + Catheter *vs*	Laminectomy + Catheter + Infusion	-3.24	0.63	**<0.001**	-5.20	-1.28
	Laminectomy + Catheter *vs*	Medullar Contusion	0.40	0.61	>0.999	-1.49	2.30
	Laminectomy + Catheter *vs*	Contusion + Catheter	-0.74	0.63	>0.999	-2.70	1.22
	Laminectomy + Catheter *vs*	Contusion + Catheter + Infusion	-0.89	0.63	>0.999	-2.85	1.06
	Laminectomy + Catheter *vs*	Laminectomy	3.13	0.61	**<0.001**	1.24	5.03
	Laminectomy + Catheter + Infusion *vs*	Medullar Contusion	3.65	0.61	**<0.001**	1.75	5.54
	Laminectomy + Catheter + Infusion *vs*	Contusion + Catheter	2.50	0.63	**0.004**	0.54	4.46
	Laminectomy + Catheter + Infusion *vs*	Contusion + Catheter + Infusion	2.35	0.63	**0.009**	0.39	4.31
	Laminectomy + Catheter + Infusion *vs*	Laminectomy	6.38	0.61	**<0.001**	4.48	8.27
	Medullar Contusion *vs*	Contusion + Catheter	-1.15	0.61	0.989	-3.04	0.75
	Medullar Contusion *vs*	Contusion + Catheter + Infusion	-1.30	0.61	0.578	-3.19	0.60
	Medullar Contusion *vs*	Laminectomy	2.73	0.58	**0.001**	0.90	4.56
	Contusion + Catheter *vs*	Contusion + Catheter + Infusion	-0.15	0.63	>0.999	-2.11	1.81
	Contusion + Catheter *vs*	Laminectomy	3.88	0.61	**<0.001**	1.98	5.77
	Contusion + Catheter + Infusion *vs*	Laminectomy	4.03	0.61	**<0.001**	2.13	5.93
Amplitude MI	Laminectomy + Catheter *vs*	Laminectomy + Catheter + Infusion	0.70	0.12	**<0.001**	0.33	1.08
	Laminectomy + Catheter *vs*	Medullar Contusion	0.74	0.12	**<0.001**	0.38	1.11
	Laminectomy + Catheter *vs*	Contusion + Catheter	0.93	0.12	**<0.001**	0.56	1.31
	Laminectomy + Catheter *vs*	Contusion + Catheter + Infusion	0.99	0.12	**<0.001**	0.61	1.37
	Laminectomy + Catheter *vs*	Laminectomy	-2.60	0.12	**<0.001**	-2.97	-2.24
	Laminectomy + Catheter + Infusion *vs*	Medullar Contusion	0.04	0.12	>0.999	-0.33	0.40
	Laminectomy + Catheter + Infusion *vs*	Contusion + Catheter	0.23	0.12	0.963	-0.15	0.61
	Laminectomy + Catheter + Infusion *vs*	Contusion + Catheter + Infusion	0.29	0.12	0.345	-0.09	0.66
	Laminectomy + Catheter + Infusion *vs*	Laminectomy	-3.31	0.12	**<0.001**	-3.67	-2.94
	Medullar Contusion *vs*	Contusion + Catheter	0.19	0.12	>0.999	-0.18	0.55
	Medullar Contusion *vs*	Contusion + Catheter + Infusion	0.25	0.12	0.629	-0.12	0.61
	Medullar Contusion *vs*	Laminectomy	-3.35	0.11	**<0.001**	-3.70	-3.00
	Contusion + Catheter *vs*	Contusion + Catheter + Infusion	0.06	0.12	>0.999	-0.32	0.43
	Contusion + Catheter *vs*	Laminectomy	-3.54	0.12	**<0.001**	-3.90	-3.17
	Contusion + Catheter + Infusion *vs*	Laminectomy	-3.59	0.12	**<0.001**	-3.96	-3.23

Bonferroni Multiple Comparisons.

**Table 2:** Comparison of histological parameters in two-by-two group comparisons.

Variable	Comparison		Z Value	*p*
Hemorrhage	Laminectomy + Catheter *vs*	Laminectomy + Catheter + Infusion	-0.41	0.679
	Laminectomy + Catheter *vs*	Medullar Contusion	-3.01	**0.003**
	Laminectomy + Catheter *vs*	Contusion + Catheter	-2.46	**0.014**
	Laminectomy + Catheter *vs*	Contusion + Catheter + Infusion	-1.23	0.220
	Laminectomy + Catheter *vs*	Laminectomy	0.52	0.605
	Laminectomy + Catheter + Infusion *vs*	Medullar Contusion	-2.80	**0.005**
	Laminectomy + Catheter + Infusion *vs*	Contusion + Catheter	-2.21	**0.027**
	Laminectomy + Catheter + Infusion *vs*	Contusion + Catheter + Infusion	-0.88	0.381
	Laminectomy + Catheter + Infusion *vs*	Laminectomy	0.97	0.333
	Medullar Contusion *vs*	Contusion + Catheter	0.59	0.557
	Medullar Contusion *vs*	Contusion + Catheter + Infusion	1.92	0.055
	Medullar Contusion *vs*	Laminectomy	3.56	**<0.001**
	Contusion + Catheter *vs*	Contusion + Catheter + Infusion	1.33	0.182
	Contusion + Catheter *vs*	Laminectomy	3.01	**0.003**
	Contusion + Catheter + Infusion *vs*	Laminectomy	1.78	0.075
Architectural Alterations	Laminectomy + Catheter *vs*	Laminectomy + Catheter + Infusion	0.32	0.749
	Laminectomy + Catheter *vs*	Medullar Contusion	-2.51	**0.012**
	Laminectomy + Catheter *vs*	Contusion + Catheter	-2.80	**0.005**
	Laminectomy + Catheter *vs*	Contusion + Catheter + Infusion	-0.92	0.360
	Laminectomy + Catheter *vs*	Laminectomy	0.70	0.483
	Laminectomy + Catheter + Infusion *vs*	Medullar Contusion	-3.06	**0.002**
	Laminectomy + Catheter + Infusion *vs*	Contusion + Catheter	-3.37	**0.001**
	Laminectomy + Catheter + Infusion *vs*	Contusion + Catheter + Infusion	-1.33	0.182
	Laminectomy + Catheter + Infusion *vs*	Laminectomy	0.43	0.667
	Medullar Contusion *vs*	Contusion + Catheter	-0.31	0.757
	Medullar Contusion *vs*	Contusion + Catheter + Infusion	1.72	0.085
	Medullar Contusion *vs*	Laminectomy	3.26	**0.001**
	Contusion + Catheter *vs*	Contusion + Catheter + Infusion	2.03	**0.042**
	Contusion + Catheter *vs*	Laminectomy	3.55	**<0.001**
	Contusion + Catheter + Infusion *vs*	Laminectomy	1.67	0.096
Necrosis	Laminectomy + Catheter *vs*	Laminectomy + Catheter + Infusion	0.21	0.834
	Laminectomy + Catheter *vs*	Medullar Contusion	-2.54	**0.011**
	Laminectomy + Catheter *vs*	Contusion + Catheter	-2.82	**0.005**
	Laminectomy + Catheter *vs*	Contusion + Catheter + Infusion	-0.70	0.482
	Laminectomy + Catheter *vs*	Laminectomy	0.39	0.695
	Laminectomy + Catheter + Infusion *vs*	Medullar Contusion	-2.97	**0.003**
	Laminectomy + Catheter + Infusion *vs*	Contusion + Catheter	-3.27	**0.001**
	Laminectomy + Catheter + Infusion *vs*	Contusion + Catheter + Infusion	-0.99	0.324
	Laminectomy + Catheter + Infusion *vs*	Laminectomy	0.21	0.834
	Medullar Contusion *vs*	Contusion + Catheter	-0.31	0.759
	Medullar Contusion *vs*	Contusion + Catheter + Infusion	1.98	**0.048**
	Medullar Contusion *vs*	Laminectomy	2.96	**0.003**
	Contusion + Catheter *vs*	Contusion + Catheter + Infusion	2.29	**0.022**
	Contusion + Catheter *vs*	Laminectomy	3.24	**0.001**
	Contusion + Catheter + Infusion *vs*	Laminectomy	1.12	0.262
Inflammatory Infiltrate	Laminectomy + Catheter *vs*	Laminectomy + Catheter + Infusion	-0.27	0.791
	Laminectomy + Catheter *vs*	Medullar Contusion	-2.43	**0.015**
	Laminectomy + Catheter *vs*	Contusion + Catheter	-2.25	**0.025**
	Laminectomy + Catheter *vs*	Contusion + Catheter + Infusion	-0.66	0.509
	Laminectomy + Catheter *vs*	Laminectomy	0.49	0.621
	Laminectomy + Catheter + Infusion *vs*	Medullar Contusion	-2.34	**0.019**
	Laminectomy + Catheter + Infusion *vs*	Contusion + Catheter	-2.14	**0.032**
	Laminectomy + Catheter + Infusion *vs*	Contusion + Catheter + Infusion	-0.43	0.669
	Laminectomy + Catheter + Infusion *vs*	Laminectomy	0.79	0.428
	Medullar Contusion *vs*	Contusion + Catheter	0.20	0.842
	Medullar Contusion *vs*	Contusion + Catheter + Infusion	1.91	0.056
	Medullar Contusion *vs*	Laminectomy	2.96	**0.003**
	Contusion + Catheter *vs*	Contusion + Catheter + Infusion	1.71	0.087
	Contusion + Catheter *vs*	Laminectomy	2.77	**0.006**
	Contusion + Catheter + Infusion *vs*	Laminectomy	1.19	0.235

Dunn Multiple Comparisons.

**Table 3:** Results of the horizontal plane comparisons between the moments evaluated in each group.

							CI (95%)
Moment/Group	Comparison		Mean Difference	Standard Deviation	*p*	Inferior	Superior
Laminectomy + Catheter	Pre-op. *vs*	2 days	4.28	2.40	>0.999	-4.70	13.27
	Pre-op. *vs*	3 weeks	6.28	2.41	>0.999	-2.74	15.29
	Pre-op. *vs*	6 weeks	0.56	2.41	>0.999	-8.45	9.58
	2 days *vs*	3 weeks	2.00	2.40	>0.999	-6.99	10.98
	2 days *vs*	6 weeks	-3.72	2.41	>0.999	-12.73	5.30
	3 weeks *vs*	6 weeks	-5.71	2.40	>0.999	-14.70	3.27
Laminectomy + Catheter + Infusion	Pre-op. *vs*	2 days	0.78	2.40	>0.999	-8.20	9.77
	Pre-op. *vs*	3 weeks	2.77	2.41	>0.999	-6.25	11.78
	Pre-op. *vs*	6 weeks	-4.05	2.41	>0.999	-13.06	4.96
	2 days *vs*	3 weeks	1.98	2.40	>0.999	-7.00	10.97
	2 days *vs*	6 weeks	-4.83	2.41	>0.999	-13.85	4.18
	3 weeks *vs*	6 weeks	-6.82	2.40	>0.999	-15.80	2.17
Medullar Contusion	Pre-op. *vs*	2 days	98.23	2.40	**<0.001**	89.24	107.21
	Pre-op. *vs*	3 weeks	98.23	2.41	**<0.001**	89.21	107.24
	Pre-op. *vs*	6 weeks	19.43	2.41	**<0.001**	10.41	28.44
	2 days *vs*	3 weeks	0.00	2.40	>0.999	-8.99	8.99
	2 days *vs*	6 weeks	-78.80	2.41	**<0.001**	-87.81	-69.79
	3 weeks *vs*	6 weeks	-78.80	2.40	**<0.001**	-87.79	-69.81
Contusion + Catheter	Pre-op. *vs*	2 days	97.04	2.40	**<0.001**	88.06	106.03
	Pre-op. *vs*	3 weeks	97.04	2.41	**<0.001**	88.03	106.06
	Pre-op. *vs*	6 weeks	16.62	2.41	**<0.001**	7.60	25.63
	2 days *vs*	3 weeks	0.00	2.40	>0.999	-8.99	8.99
	2 days *vs*	6 weeks	-80.43	2.41	**<0.001**	-89.44	-71.41
	3 weeks *vs*	6 weeks	-80.43	2.40	**<0.001**	-89.41	-71.44
Contusion + Catheter + Infusion	Pre-op. *vs*	2 days	96.51	2.40	**<0.001**	87.53	105.50
	Pre-op. *vs*	3 weeks	96.51	2.41	**<0.001**	87.50	105.53
	Pre-op. *vs*	6 weeks	24.78	2.41	**<0.001**	15.77	33.80
	2 days *vs*	3 weeks	0.00	2.40	>0.999	-8.99	8.99
	2 days *vs*	6 weeks	-71.73	2.41	**<0.001**	-80.74	-62.72
	3 weeks *vs*	6 weeks	-71.73	2.40	**<0.001**	-80.72	-62.74
Laminectomy	Pre-op. *vs*	2 days	0.88	2.40	>0.999	-8.10	9.87
	Pre-op. *vs*	3 weeks	0.62	2.41	>0.999	-8.40	9.63
	Pre-op. *vs*	6 weeks	2.92	2.41	>0.999	-6.09	11.94
	2 days *vs*	3 weeks	-0.27	2.40	>0.999	-9.25	8.72
	2 days *vs*	6 weeks	2.04	2.41	>0.999	-6.97	11.05
	3 weeks *vs*	6 weeks	2.31	2.40	>0.999	-6.68	11.29

Bonferroni Multiple Comparisons.

**Table 4:** Results of the BBB score comparisons between the moments evaluated in each group.

							CI (95%)
Moment/Group	Comparison		Mean Difference	Standard Deviation	*p*	Inferior	Superior
Laminectomy + Catheter	2 days *vs*	1 week	-0.19	0.31	>0.999	-1.42	1.04
	2 days *vs*	2 weeks	-0.38	0.40	>0.999	-2.00	1.25
	2 days *vs*	3 weeks	-0.94	0.46	>0.999	-2.80	0.92
	2 days *vs*	4 weeks	-1.06	0.50	>0.999	-3.08	0.95
	2 days *vs*	5 weeks	-1.19	0.53	>0.999	-3.31	0.94
	2 days *vs*	6 weeks	-1.19	0.55	>0.999	-3.39	1.01
	1 week *vs*	2 weeks	-0.19	0.31	>0.999	-1.42	1.04
	1 week *vs*	3 weeks	-0.75	0.40	>0.999	-2.37	0.87
	1 week *vs*	4 weeks	-0.88	0.46	>0.999	-2.74	0.99
	1 week *vs*	5 weeks	-1.00	0.50	>0.999	-3.02	1.02
	1 week *vs*	6 weeks	-1.00	0.53	>0.999	-3.12	1.12
	2 weeks *vs*	3 weeks	-0.56	0.31	>0.999	-1.79	0.67
	2 weeks *vs*	4 weeks	-0.69	0.40	>0.999	-2.31	0.94
	2 weeks *vs*	5 weeks	-0.81	0.46	>0.999	-2.67	1.05
	2 weeks *vs*	6 weeks	-0.81	0.50	>0.999	-2.83	1.20
	3 weeks *vs*	4 weeks	-0.13	0.31	>0.999	-1.36	1.11
	3 weeks *vs*	5 weeks	-0.25	0.40	>0.999	-1.87	1.37
	3 weeks *vs*	6 weeks	-0.25	0.46	>0.999	-2.11	1.61
	4 weeks *vs*	5 weeks	-0.13	0.31	>0.999	-1.36	1.11
	4 weeks *vs*	6 weeks	-0.13	0.40	>0.999	-1.75	1.50
	5 weeks *vs*	6 weeks	0.00	0.31	>0.999	-1.23	1.23
Laminectomy + Catheter + Infusion	2 days *vs*	1 week	-0.25	0.31	>0.999	-1.48	0.98
	2 days *vs*	2 weeks	-0.38	0.40	>0.999	-2.00	1.25
	2 days *vs*	3 weeks	-0.13	0.46	>0.999	-1.99	1.74
	2 days *vs*	4 weeks	-0.88	0.50	>0.999	-2.89	1.14
	2 days *vs*	5 weeks	-1.06	0.53	>0.999	-3.19	1.06
	2 days *vs*	6 weeks	-1.13	0.55	>0.999	-3.33	1.08
	1 week *vs*	2 weeks	-0.13	0.31	>0.999	-1.36	1.11
	1 week *vs*	3 weeks	0.13	0.40	>0.999	-1.50	1.75
	1 week *v*s	4 weeks	-0.63	0.46	>0.999	-2.49	1.24
	1 week *vs*	5 weeks	-0.81	0.50	>0.999	-2.83	1.20
	1 week *vs*	6 weeks	-0.88	0.53	>0.999	-3.00	1.25
	2 weeks *vs*	3 weeks	0.25	0.31	>0.999	-0.98	1.48
	2 weeks *vs*	4 weeks	-0.50	0.40	>0.999	-2.12	1.12
	2 weeks *vs*	5 weeks	-0.69	0.46	>0.999	-2.55	1.17
	2 weeks *vs*	6 weeks	-0.75	0.50	>0.999	-2.77	1.27
	3 weeks *vs*	4 weeks	-0.75	0.31	>0.999	-1.98	0.48
	3 weeks *vs*	5 weeks	-0.94	0.40	>0.999	-2.56	0.69
	3 weeks *vs*	6 weeks	-1.00	0.46	>0.999	-2.86	0.86
	4 weeks *vs*	5 weeks	-0.19	0.31	>0.999	-1.42	1.04
	4 weeks *vs*	6 weeks	-0.25	0.40	>0.999	-1.87	1.37
	5 weeks *vs*	6 weeks	-0.06	0.31	>0.999	-1.29	1.17
Medullar Contusion	2 days *vs*	1 week	0.00	0.31	>0.999	-1.23	1.23
	2 days *vs*	2 weeks	-2.25	0.40	**<0.001**	-3.87	-0.63
	2 days *vs*	3 weeks	-4.69	0.46	**<0.001**	-6.55	-2.83
	2 days *vs*	4 weeks	-6.25	0.50	**<0.001**	-8.27	-4.23
	2 days *vs*	5 weeks	-6.38	0.53	**<0.001**	-8.50	-4.25
	2 days *vs*	6 weeks	-6.88	0.55	**<0.001**	-9.08	-4.68
	1 week *vs*	2 weeks	-2.25	0.31	**<0.001**	-3.48	-1.02
	1 week *vs*	3 weeks	-4.69	0.40	**<0.001**	-6.31	-3.06
	1 week *vs*	4 weeks	-6.25	0.46	**<0.001**	-8.11	-4.39
	1 week *vs*	5 weeks	-6.38	0.50	**<0.001**	-8.39	-4.36
	1 week *vs*	6 weeks	-6.88	0.53	**<0.001**	-9.00	-4.75
	2 weeks *vs*	3 weeks	-2.44	0.31	**<0.001**	-3.67	-1.21
	2 weeks *vs*	4 weeks	-4.00	0.40	**<0.001**	-5.62	-2.38
	2 weeks *vs*	5 weeks	-4.13	0.46	**<0.001**	-5.99	-2.27
	2 weeks *vs*	6 weeks	-4.63	0.50	**<0.001**	-6.64	-2.61
	3 weeks *vs*	4 weeks	-1.56	0.31	**<0.001**	-2.79	-0.33
	3 weeks *vs*	5 weeks	-1.69	0.40	**0.025**	-3.31	-0.06
	3 weeks *vs*	6 weeks	-2.19	0.46	**0.002**	-4.05	-0.33
	4 weeks *vs*	5 weeks	-0.13	0.31	>0.999	-1.36	1.11
	4 weeks *vs*	6 weeks	-0.63	0.40	>0.999	-2.25	1.00
	5 weeks *vs*	6 weeks	-0.50	0.31	>0.999	-1.73	0.73
Contusion + Catheter	2 days *vs*	1 week	-0.13	0.31	>0.999	-1.36	1.11
	2 days *vs*	2 weeks	-2.38	0.40	**<0.001**	-4.00	-0.75
	2 days *vs*	3 weeks	-4.63	0.46	**<0.001**	-6.49	-2.77
	2 days *vs*	4 weeks	-5.13	0.50	**<0.001**	-7.14	-3.11
	2 days *vs*	5 weeks	-5.25	0.53	**<0.001**	-7.37	-3.13
	2 days *vs*	6 weeks	-6.13	0.55	**<0.001**	-8.33	-3.93
	1 week *vs*	2 weeks	-2.25	0.31	**<0.001**	-3.48	-1.02
	1 week *vs*	3 weeks	-4.50	0.40	**<0.001**	-6.12	-2.88
	1 week *vs*	4 weeks	-5.00	0.46	**<0.001**	-6.86	-3.14
	1 week *vs*	5 weeks	-5.13	0.50	**<0.001**	-7.14	-3.11
	1 week *vs*	6 weeks	-6.00	0.53	**<0.001**	-8.12	-3.88
	2 weeks *vs*	3 weeks	-2.25	0.31	**<0.001**	-3.48	-1.02
	2 weeks *vs*	4 weeks	-2.75	0.40	**<0.001**	-4.37	-1.13
	2 weeks *vs*	5 weeks	-2.88	0.46	**<0.001**	-4.74	-1.02
	2 weeks *vs*	6 weeks	-3.75	0.50	**<0.001**	-5.77	-1.73
	3 weeks *vs*	4 weeks	-0.50	0.31	>0.999	-1.73	0.73
	3 weeks *vs*	5 weeks	-0.63	0.40	>0.999	-2.25	1.00
	3 weeks *vs*	6 weeks	-1.50	0.46	>0.999	-3.36	0.36
	4 weeks *vs*	5 weeks	-0.13	0.31	>0.999	-1.36	1.11
	4 weeks *vs*	6 weeks	-1.00	0.40	>0.999	-2.62	0.62
	5 weeks *vs*	6 weeks	-0.88	0.31	>0.999	-2.11	0.36
Contusion + Catheter + Infusion	2 days *vs*	1 week	-0.94	0.31	>0.999	-2.17	0.29
	2 days *vs*	2 weeks	-2.13	0.40	**<0.001**	-3.75	-0.50
	2 days *vs*	3 weeks	-2.50	0.46	**<0.001**	-4.36	-0.64
	2 days *vs*	4 weeks	-4.13	0.50	**<0.001**	-6.14	-2.11
	2 days *vs*	5 weeks	-4.63	0.53	**<0.001**	-6.75	-2.50
	2 days *vs*	6 weeks	-4.75	0.55	**<0.001**	-6.95	-2.55
	1 week *vs*	2 weeks	-1.19	0.31	0.092	-2.42	0.04
	1 week *vs*	3 weeks	-1.56	0.40	0.094	-3.19	0.06
	1 week *vs*	4 weeks	-3.19	0.46	**<0.001**	-5.05	-1.33
	1 week *vs*	5 weeks	-3.69	0.50	**<0.001**	-5.70	-1.67
	1 week *vs*	6 weeks	-3.81	0.53	**<0.001**	-5.94	-1.69
	2 weeks *vs*	3 weeks	-0.38	0.31	>0.999	-1.61	0.86
	2 weeks *vs*	4 weeks	-2.00	0.40	**0.001**	-3.62	-0.38
	2 weeks *vs*	5 weeks	-2.50	0.46	**<0.001**	-4.36	-0.64
	2 weeks *vs*	6 weeks	-2.63	0.50	**<0.001**	-4.64	-0.61
	3 weeks *vs*	4 weeks	-1.63	0.31	**<0.001**	-2.86	-0.39
	3 weeks *vs*	5 weeks	-2.13	0.40	**<0.001**	-3.75	-0.50
	3 weeks *vs*	6 weeks	-2.25	0.46	**0.001**	-4.11	-0.39
	4 weeks *vs*	5 weeks	-0.50	0.31	>0.999	-1.73	0.73
	4 weeks *vs*	6 weeks	-0.63	0.40	>0.999	-2.25	1.00
	5 weeks *vs*	6 weeks	-0.13	0.31	>0.999	-1.36	1.11
Laminectomy	2 days *vs*	1 week	0.00	0.31	>0.999	-1.23	1.23
	2 days *vs*	2 weeks	0.00	0.40	>0.999	-1.62	1.62
	2 days *vs*	3 weeks	0.00	0.46	>0.999	-1.86	1.86
	2 days *vs*	4 weeks	0.00	0.50	>0.999	-2.02	2.02
	2 days *vs*	5 weeks	0.00	0.53	>0.999	-2.12	2.12
	2 days *vs*	6 weeks	0.00	0.55	>0.999	-2.20	2.20
	1 week *vs*	2 weeks	0.00	0.31	>0.999	-1.23	1.23
	1 week *vs*	3 weeks	0.00	0.40	>0.999	-1.62	1.62
	1 week *vs*	4 weeks	0.00	0.46	>0.999	-1.86	1.86
	1 week *vs*	5 weeks	0.00	0.50	>0.999	-2.02	2.02
	1 week *vs*	6 weeks	0.00	0.53	>0.999	-2.12	2.12
	2 weeks *vs*	3 weeks	0.00	0.31	>0.999	-1.23	1.23
	2 weeks *vs*	4 weeks	0.00	0.40	>0.999	-1.62	1.62
	2 weeks *vs*	5 weeks	0.00	0.46	>0.999	-1.86	1.86
	2 weeks *vs*	6 weeks	0.00	0.50	>0.999	-2.02	2.02
	3 weeks *vs*	4 weeks	0.00	0.31	>0.999	-1.23	1.23
	3 weeks *vs*	5 weeks	0.00	0.40	>0.999	-1.62	1.62
	3 weeks *vs*	6 weeks	0.00	0.46	>0.999	-1.86	1.86
	4 weeks *vs*	5 weeks	0.00	0.31	>0.999	-1.23	1.23
	4 weeks *vs*	6 weeks	0.00	0.40	>0.999	-1.62	1.62
	5 weeks *vs*	6 weeks	0.00	0.31	>0.999	-1.23	1.23

Bonferroni Multiple Comparisons.
